# The Effectiveness of Psychosocial Interventions with Cancer Patients: An Integrative Review of the Literature (2006–2011)

**DOI:** 10.5402/2011/638218

**Published:** 2011-11-16

**Authors:** Bonnie Raingruber

**Affiliations:** California State University, Sacramento and University of California, Davis, Sacramento, CA 95817, USA

## Abstract

*Background*. Previous integrative literature reviews and meta-analyses have yielded conflicting results regarding the effectiveness of psychosocial interventions for cancer patients. *Methods*. An integrative review of the literature focused on 19 randomized, controlled trials (2006–2011) was completed to examine the effectiveness of psychosocial interventions for cancer patients. Eligibility criteria: Inclusion criteria were the study was an English language randomized controlled clinical trial. *Results*. Seven studies involved nurses. Eleven studies resulted in positive outcomes. Overall, study quality was limited. In eight studies the intervention was not adequately described, 7 studies did not contain a hypothesis, 4 did not include clear eligibility criteria, 10 studies did not randomize appropriately, 9 did not list recruitment dates, 11 did not include a power analysis, 14 did not include blinded patients or data collectors, 11 did not use an intent-to-treat analysis, 10 did not clarify reasons for drop outs, and 11 did not discuss treatment fidelity. *Conclusions*. Future studies should build on previous findings, use comparable outcome measures, and adhere to standards of quality research. Qualitative studies are needed to determine what cancer patients of varied ages, cancer stages, and racial/ethnic backgrounds believe would be an effective intervention to manage their psychosocial needs.

## 1. Introduction

Therapeutic communication and supportive therapies are mainstays of mental health nursing practice. These psychosocial interventions are valuable adjuncts to physical treatment for individuals who have been diagnosed with cancer. It has been determined that 33% of individuals diagnosed with cancer experience severe psychological distress and up to 70% exhibit some degree of anxiety and depression [1–3]. Relationships, work life, and sense of self are all impacted by a cancer diagnosis [4].

Excellent care must include interventions that focus on the informational and psychosocial needs of patients [5]. Facilitating emotional expression helps to modulate distress and enhance coping abilities [6]. Psychosocial interventions including therapeutic communication have been used with success to minimize stress, improve quality of life, treat depression, and support cancer patients throughout the course of their diagnosis and recovery [1, 7]. 

A national symposium organized by the Agency for Health Care Research and Quality, the National Cancer Institute, and experts in the field concluded that communication and interventions targeting psychosocial issues were among the 8 key domains that are of vital importance in cancer care. These domains highlight the challenges that cancer patients face in coping with their emotions and navigating life disruptions associated with their treatment [8]. 

## 2. Previous Meta-Analyses and Integrative Reviews

A number of meta-analysis and reviews of the literature have focused on the effectiveness of psychosocial interventions with cancer patients. A meta-analysis conducted by Meyer and Mark [9] concluded that relaxation and behavioral modification improved functional adaptation and symptom control but did not affect medical outcomes. Newell et al. [10] reported that group-based and individual therapy, educational interventions, and guided imagery were effective with cancer patients. Most of these interventions were provided by a psychiatrist or psychologist, not nurses, and involved at least six contact hours. Barsevick et al. [11] conducted a systematic review of 36 studies to conclude psychoeducational interventions as well as behavior therapy reduced depression in cancer patients.

Rehse and Pukrop [12] conducted a meta-analysis of 37 controlled studies and found that psychosocial interventions improve quality of life among cancer patients. Studies between 1970 and 1999 published in English and German were included. Most studies were conducted in university settings and included primarily well-educated white participants. Females were twice as likely to be included given the frequency of breast cancer diagnoses. Only one outcome point was considered in the meta-analysis. An overall moderate effect sized of 0.31 was reported (even when Rosenthal's fail safe method was used to estimate unpublished studies). The duration of the intervention (greater than 15 weeks) was the only significant predictor that remained after controlling for other variables. The authors suggested that educational programs were more effective because other psychosocial interventions consisted of heterogeneous techniques while education was more consistently defined and implemented across studies.

Uitterhoeve et al. [13] reviewed the literature to examine the effectiveness of psychosocial interventions (specifically cognitive behavioral therapy) on quality of life for individuals diagnosed with advanced cancer. Thirteen studies published between 1990 and 2002 were critiqued. Nurses delivered the majority of interventions (*n* = 8 studies). In 12 of the 13 trials positive quality of life changes including improved levels of depression were seen. 

Also in 2004, Chow et al. [14] conducted a meta-analysis on randomized controlled trials published between 1966 and 2002 and reported that psychosocial intervention did not prolong survival among cancer patients. Only 8 trials were reviewed, patients with differing types of metastatic illness were included, and trials with short follow-up times were reviewed, all of which limited the conclusiveness of the results. 

Williams and Dale [15] published a systematic literature review that questioned whether psychotherapeutic interventions are effective in reducing depression among cancer patients. Their review included 18 trials published between 1995 and 2005. All studies provided clear descriptions of the intervention and had representative samples. Sixteen of the studies reported reasons patients gave for dropping out of the study. Several trials found cognitive behavioral therapy and social support reduced symptoms of depression. The major flaw identified by the author was that numerous studies were single-centre trials that did not monitor for use of other interventions that could have confounded the results. 

Also in 2006 Osborn et al. [16] published a meta-analysis of the effectiveness of psychosocial interventions for depression, anxiety, and quality of life among cancer survivors. Included were 15 randomized controlled trials published between 1993 and 2004. Cognitive behavioral therapy was described as being effective in reducing depression, minimizing anxiety, and improving quality of life. Individual interventions were more effective than group interventions.

Jacobsen and Jim [17] summarized the results of systematic reviews and meta-analysis of the effects of psychosocial intervention on anxiety and depression among cancer patients. Randomized controlled trials between 1980 and 2003 were examined. Fourteen publications made reference to anxiety and 6 reported positive outcomes from psychosocial interventions. Nine studies reported psychosocial interventions were effective in improving symptoms of depression. The authors critiqued numerous methodological issues in the studies that were reviewed.

Edwards and Hulber-Williams [18] published a review of psychological interventions for women with metastatic breast cancer on psychological and survival outcomes. Randomized clinical trials published between 1966 and 2006 were examined. Five studies (cognitive behavioral and supportive-expressive) used a group format and showed limited evidence of benefit. Edwards and Hulber-Williams argued for standardization of outcome instruments, inclusion of cost effectiveness analysis and suggested interventions be designed based on preferences of individuals with cancer. 

This integrative review of the literature was conducted to examine whether recent research has shown psychosocial interventions to be effective with cancer patients. The review summarizes randomized controlled studies and integrative reviews that have been published between 2006 and 2010. During that period strides were made in medical treatment and knowledge of the influence of cortisol levels [19]. Patient attitudes toward psychosocial intervention may have shifted, reimbursement policies have been modified, and types of practitioners involved in provision of care have changed. Insufficient guidance currently exists for health care providers and researchers regarding the most effective type of psychosocial care [20]. It is necessary to understand whether, how, and why psychosocial interventions are effective with individuals who have been diagnosed with cancer. It is also critical to examine published studies if we are to design additional research that builds in a progressive manner on existing studies. If gaps in the literature are narrowed by successive refinements of psychosocial interventions and research methods we will have a more coherent research base from which to design future clinical interventions and research.

## 3. Methods

An integrative review of the literature, not a meta-analysis, designed to determine effect size was conducted. Electronic searches of Pub Med, Cinahl, and the Cochrane Library between 1/1/2006 (since the last published integrative literature review and meta-analysis) and 2/23/2011 were completed using the key words listed in Table 1. The type of research design was not specified in this search. This was done to identify as many relevant articles as possible and to avoid use of the search terms “randomized controlled trial” that might have resulted in missing relevant articles. Patients with cancer of any type or stage were included, and the review was not limited to a specific outcome measure such as depression or quality of life. This was done because given the small number of studies located (*n* = 19) it was not possible to target specific outcome measures such as depression or quality of life although several studies documented decreased depression and decreased anxiety [7, 21–23], improved quality of life [23, 24], and enhanced functional status/well-being [25, 26]. References of all 19 papers were examined using the snowball method to identify additional studies. No studies that met the inclusion criteria were identified using the snowball method. No unpublished studies were located.

Of the above 953 articles initially identified, 42 were located with more than one search term and eliminated for being duplicative. Eight hundred forty-four studies were eliminated for not being randomized controlled trials. Only 27 (19 included +8 excluded) studies identified using search terms were randomized controlled trials. Inclusion criteria were that the study was a randomized controlled clinical trial published in English, involving adult cancer patients living at home who were over the age of 18 (*n* = 19). Randomized controlled trials that used volunteers to provide the intervention, focused on cancer patients not living at home, included patient spouses in the intervention, or included pharmacological, physical therapy, and/or complementary and alternative interventions were excluded. 

Eight randomized controlled trials were eliminated for not meeting inclusion criteria. One study was excluded as it was a composite of hypnosis (an alternative treatment) and supportive-expressive group therapy [27]. One study was eliminated because it included an intervention that included conditioning exercises provided by a physical therapist [28]. Another study was eliminated because it included a couple-focused group intervention [29]. Two studies were excluded because they combined psychosocial interventions along with use of medications in studies involving cancer patients [1, 30]. One study was excluded because it focused on elderly individuals with cancer living in care homes rather than at home [21]. One study was eliminated because it used volunteers to provide the intervention [24]. One study was eliminated because it reported the qualitative results obtained from a randomized controlled trial [20]. A data extraction form which is labeled Table 2 was developed based on Consolidated Standards of Reporting Trials (-CONSORT) guidelines [22] and used to summarize the included studies and to rank their quality.

## 4. Results

Nineteen randomized controlled trials focusing on therapeutic communication and supportive interventions were grouped into four main categories: cognitive behavioral interventions, supportive interventions, group interventions, and telephone-assisted interventions. Of the 19 studies reviewed 11 studies demonstrated positive findings [7, 23, 25, 26, 31, 32, 34, 35, 38, 40, 41] while an additional 4 demonstrated positive findings only after a post hoc analysis was completed [5, 19, 33, 36]. Findings from each category of article are described below.

### 4.1. Cognitive Behavioral Interventions

Cognitive behavioral interventions were based on the belief that how individuals view situations influences their emotional response and problem-solving ability. Cognitive behavioral interventions are summarized in Table 3. Antoni et al. [7, 32] reported different outcome measures from a single study of female breast cancer patients. In one publication the intervention group had lower anxiety and cortisol and higher cytokine production than the control group [32]. In the second article, a greater reduction in cancer-specific thought intrusion, anxiety, and emotional distress was described in the cognitive behavioral intervention group [7]. Miller et al. [25] reported quality of life improved for patients with colorectal, head/neck, lung, and breast cancer who participated in 8 sessions of a cognitive behavioral intervention. Pitceathly et al. [26] found high-risk patients with breast, lymphoma, or gynecological cancer who received cognitive behavioral therapies were less likely to become depressed or anxious.

### 4.2. Supportive Interventions

Randomized controlled trials focused on supportive psychosocial interventions that included listening, validation, stress management, problem solving, and education related to the diagnosis. Interventions in this category varied substantially including (1) a one-time meeting with a psychologist for clients with gynecological cancer [41], (2) two communication skills training sessions with nurses for clients with gastric, colorectal, or breast cancer [40], (3) a coping and communication-enhancing intervention, supportive counseling or usual care intervention offered in six hour-long sessions with a therapist for patients with gynecological cancers [35], and (4) an educational and stress management intervention lasting 2 hours for patients with melanoma [39]. A coping and communication support intervention provided by mental health nurses who offered an in-home meeting and a follow-up telephone contact for patients with lung, pancreatic, or liver cancer [42] is discussed in the telephone intervention section even though it is both supportive and telephone based. Supportive interventions are summarized in Table 4. 

Although each of the supportive interventions resulted in improved outcomes, each study used a different type of supportive care, was implemented by varied types of practitioners, relied on a spectrum of different outcome measures, and included clients with several types and stages of cancer. The use of varied interventions as well as the limitations listed in Table 2 made it difficult to make any definitive conclusions about these randomized, controlled trials.

### 4.3. Group Interventions

Several researchers conducted randomized controlled trials to explore the effectiveness of group approaches that included psychosocial interventions for individuals diagnosed with cancer. Group interventions are summarized in Table 5. As with the supportive approaches the actual group interventions that were used differed significantly. Interventions for individuals with breast cancer included (1) 26 group sessions offered over a one-year period led by clinical psychologists [23, 31, 38], (2) 1 year of group therapy offered by psychologists, psychiatrists, or social workers [19], and (3) weekly supportive expressive group therapy and education lead by psychiatrists, psychologists, and social workers [36]. The length and number of sessions offered, the professionals involved, and the outcome measures used in these studies varied.

One meta-analysis was also published on the effectiveness of cancer support groups. This meta-analysis covered the years of 1981 to 2001 and included 20 randomized controlled trials. Results indicated that support group participation results in decreased depression and anxiety, increased illness adaptation, improved quality of life, and enhanced marital relationships. Group interventions did not impact survival. Forty-five percent of principal investigators were psychologists, 35% were nurses, and 20% were physicians. Seventy % of studies involved women, 65% focused on women with breast cancer, 96 % offered group sessions on a weekly basis, and a cognitive-behavioral model was used in 92% of studies. Twelve studies focused on depression, 11 on anxiety, 6 on quality of life, 8 on adaptation to illness, 3 on survival, and 2 on marital relationships [45].

### 4.4. Telephone-Assisted Interventions

Six randomized controlled trials examined support provided by telephone. These telephone assisted interventions are summarized in Table 6. Telephone-assisted interventions typically included in-person contacts to establish rapport, followed by periodic telephone contact and support [5, 6, 34, 37, 42]. Kravitz et al. [34] and Girgis et al. [33] compared telephone-based care to care provided by a physician. Two studies included family members in the initial visit [5, 6]. In-person contacts among the telephone studies ranged from 1 session [5, 6] to 6 to 8 sessions [37]. In these studies challenges such as symptoms limiting travel, geography, and intervention-associated costs provided the rationale for using telephone-assisted support [20]. Telephone contact was used because it can be a flexible way to provide timely intervention during a stressful period [6]. 

The studies by Rose et al. [6, 42] were based on the same sample, collected at 6 weeks [6] showing middle-aged patients had more problems communicating with family members and at 2 months [42] showing middle-aged patients averaged more contacts per month compared to individuals aged 61 to 80. 

Aranda et al. [5] used a brief, nurse-delivered visit followed by one phone call. The intervention was not more effective than usual care. The authors postulated that the intervention was too brief to be effective. When the results were analyzed only with women with high needs the intervention was effective in reducing psychological and emotional needs. 

Walker et al. [37] randomized 200 individuals with lung cancer to a usual care or depression care intervention. Nurses offered 6 to 8 in-person sessions followed by monthly telephone contact. Results are pending as data collection is continuing until June of 2011.

Kravitz et al. [34] compared telephone intervention with care provided by a physician to find that short-term outcomes measured at 2 weeks improved although the long-term outcomes at 6 and 12 weeks were not improved. Girgis et al. [33] compared telephone caseworkers (oncology nurses) to a general practitioner model to find no overall intervention effect.

Additional research on the frequency, timing of in relation to diagnosis, and length of telephone support is needed to determine what level of intervention is needed. In addition, whether regional and generational differences exist in response to telephone support needs to be examined [20].

### 4.5. Overall Conclusions Regarding the Effectiveness of Psychosocial Interventions

Overall, 11 of the 19 studies included in this integrative review showed positive results [7, 23, 25, 26, 31, 32, 34, 35, 38, 40, 41], and an additional 4 showed positive results [5, 19, 33, 36] based on a post hoc analysis. Consistent outcomes that were affected by more than one study in this integrative review included decreased depression [19, 26, 35, 41], decreased anxiety [7, 26, 32, 41], improved quality of life [25, 41], and enhanced functional status/well-being [31, 38]. These positive results demonstrating the effectiveness of psychosocial interventions are consistent with positive results from previous meta-analyses [9, 12, 16, 17, 45] and integrative reviews [11, 13]. In contrast, one previous integrative review reported that psychosocial interventions do not improve depression [15] while two integrative reviews reported psychosocial interventions do not prolong life [14, 18]. This outcome of not increasing survival time is consistent with findings from this integrative review that showed survival time in two studies did not increase in the group receiving psychosocial interventions [19, 36] while one study reported a decreased risk of cancer reoccurrence in the group receiving psychosocial intervention [9]. Several authors of meta-analyses and integrative reviews have stressed the need for greater consistency within the research trajectory in this area in order to report conclusively that psychosocial interventions are effective [15, 18].

### 4.6. A Summary Regarding the Quality of Studies Reviewed

Variations among outcome measures, treatment conditions (cancer stage, treatment type, treatment location, and group/individual approach), age, racial/ethnic background, and methodological quality made it difficult to draw definitive conclusions from this integrative review. What is clear is that both clinicians and researchers would benefit from future studies which build on previous findings, use comparable outcome measures, and adhere to standards of quality research such as those identified in the CONSORT guidelines [22]. It is important to identify what types and duration of intervention are effective for what population. As Jacobsen and Jim [17] commented “psychosocial care that is ineffective may be worse than no care at all” (p. 214). All of the 19 studies reviewed included detailed information about the analysis phase of their research. But as is evident in Table 2, not all of the studies reviewed included all of the quality measures that could be expected in a randomized, controlled clinical trial. Fewer studies (8 out of 19) included a power analysis, fewer studies addressed treatment fidelity (8 out of 19), fewer studies (9 out of 19) specified the method of randomization, fewer studies (5 out of 19) discussed blinding, fewer studies (9 out of 19) described reasons for dropping out of the study, and fewer studies (8 out of 19) used an intent-to-treat analysis.

Researchers need to include the quality measures listed in the CONSORT guidelines [22], incorporate cancer-specific outcomes measures, and explore whether certain interventions are more effective in select racial/ethnic or age groups. Additional research in needed to clarify if differences in time of diagnosis, cancer stage, the influence of age, or geographic area impact the effectiveness of psychosocial interventions. 

It is useful to examine individual study quality by comparing a perfect score of 13 based on allocation of 1 point based on whether (1) a hypothesis was provided, (2) eligibility criteria are clear, (3) the intervention is described in sufficient detail for replication, (4) treatment fidelity is discussed, (5) a power analysis was provided, (6) the method of randomization was clear, (7) blinding was discussed, (8) reasons for dropouts were given, (9) an intent-to-treat analysis was used, (10) recruitment dates were specified, (11) study limitations were described, (12) funding sources were listed, and (13) a trial registry was mentioned. These 13 criteria included in the CONSORT guidelines [22] and summarized in Table 2 provide guidance when ranking study quality. Using these 13-point criteria Pitceathy et al. [26] and Walker et al. [37] received high scores of 11, followed by a score of 9 for Kravitz et al. [34], Spiegel et al. [36], and Kissane et al. [19] and by a score of 8 achieved by Andersen et al. [23] and Girgis et al. [33]. A midrange score of 7 is observed for Andersen et al. [31], Antoni et al. [32], Manne et al. [35], and Fukui et al. [40] while a score of 6 is seen for Antoni et al. [7] and Miller et al. [25]. Lower quality scores of 5 are observed for Aranda et al. [5], Andersen et al. [38], Boesen et al. [39], and Powell et al. [41]. The lowest quality scores of 3 for Rose et al. [42] and 4 for Rose et al. [6] are also evident. It is, however, important to remember that not all of the above-mentioned scores obtained from the CONSORT guidelines [22] are of strictly equivalent value for determining study quality.

Only eight studies [19, 26, 32, 34, 36–38, 40] did an adequate job of assuring treatment fidelity including (1) basing interventions on theory, (2) offering standardized training to those implementing the intervention, (3) monitoring to ensure interventions were consistently provided, (4) reinforcing training to avoid loss of skills or variation in approach, and (5) minimizing contact between treatment and control groups [46]. 

Too few studies have included mental health nurses or been designed by mental health nurses [6, 42]. Although it is a challenge given current economic realities, there is a need for mental health nurses to locate funding for randomized clinical trials and to focus on the effectiveness of mainstay interventions within mental health nursing practice, namely, therapeutic communication and supportive care in minimizing depressive symptoms and improving quality of life measures among individuals who have been diagnosed with cancer.

Eleven studies documented positive outcomes from psychological interventions [7, 23, 25, 26, 31, 32, 34, 35, 38, 40, 41] and an additional 4 showed positive results [5, 19, 33, 36] based on ad hoc analysis. Data collection is continuing for 2 studies [6, 37]. Additional research is needed because the literature continues to be full of methodological gaps and discrepancies. As Edwards et al. commented [18] it is difficult to integrate the results of randomized, controlled trials because of the variability of interventions, age groups, ethnic/racial backgrounds, cancer stages, outcome measures, and methodological quality. It is critical to increase the number of qualitative studies to determine what sort of intervention cancer patients of varied ages, ethnic/racial backgrounds, and cancer stages feel would meet their psychosocial needs. When those perspectives have been assessed researchers can design more effective randomized clinical trials that help close existing gaps in the literature.

## Figures and Tables

**Table 1:** Search terms used.

Keywords	Total articles located
Therapeutic communication and cancer	96
Psychological support and cancer	375
Psychosocial and cancer	174
Communication and cancer	308

**Table 2:** Study quality.

Quality measure	Author	Type of study
A hypothesis is provided (1 point)	Andersen et al. [31], Andersen et al. [23], Antoni et al. [7], Antoni et al. [32], Aranda et al. [5], Girgis et al. [33], Kravitz et al. [34], Manne et al. [35], Miller et al. [25], Pitceathy et al. [26], Spiegel et al. [36], Walker et al. [37].	4 Cognitive behavioral 1 Supportive 3 Group 4 Telephone

A hypothesis is not provided	Andersen et al. [38], Boesen et al. [39], Fukui et al. [40], Kissane et al. [19], Powell et al. [41] Rose et al. [6], Rose et al. [42].	3 Supportive 2 Group 2 Telephone

Eligibility criteria are clear (1 point)	Andersen et al. [31], Andersen et al. [38], Andersen et al. [23], Antoni et al. [7], Antoni et al. [32], Boesen et al. [39], Fukui et al. [40], Girgis et al. [33], Kissane et al. [19], Kravitz et al. [34], Manne et al. [35], Rose et al. [6], Rose et al. [42], Spiegel et al. [36], Walker et al. [37].	2 Cognitive behavioral 3 Supportive 5 Group 5 Telephone

Eligibility criteria are not clear	Aranda et al. [5], Miller et al. [25], Pitceathy et al. [26], Powell et al. [41].	2 Cognitive behavioral 1 Supportive 1 Telephone

The intervention is described with sufficient detail it could be replicated (1 point)	Andersen et al. [31], Andersen et al. [38], Andersen et al. [23], Antoni et al. [7], Antoni et al. [32], Aranda et al. [5], Kissane et al. [19], Kravitz et al. [34], Pitceathy et al. [26], Rose et al. [6], Walker et al. [37].	3 Cognitive behavioral 4 Group 4 Telephone

The intervention is not described with sufficient detail to allow for replication	Boesen et al. [39], Fukui et al. [40], Girgis et al. [33], Manne et al. [35], Miller et al. [25], Powell et al. [41], Rose et al. [42], Spiegel et al. [36].	1 Cognitive behavioral 4 Supportive 1 Group 2 Telephone

Treatment fidelity is discussed (1 point)	Andersen et al. [38], Antoni et al. [32], Fukui et al. [40], Kissane et al. [19], Kravitz et al. [34], Pitceathy, et al. [26], Spiegel et al. [36], Walker et al. [37].	2 Cognitive behavioral 1 Supportive 3 Group 2 Telephone

Treatment fidelity is not addressed	Andersen et al. [31], Andersen et al. [23], Antoni et al. [7], Aranda et al. [5], Boesen et al. [39], Girgis et al. [33], Manne et al. [35], Miller et al. [25], Powell et al. [41], Rose et al. [6], Rose et al. [42].	2 Cognitive behavioral 3 Supportive 2 Group 4 Telephone

A power analysis was provided to establish the sample size (1 point)	Andersen et al. [23], Girgis et al. [33], Manne et al. [35], Miller et al. [25], Pitceathy et al. [26], Powell et al. [41], Spiegel et al. [36], Walker et al. [37].	2 Cognitive behavioral 2 Supportive 2 Group 2 Telephone

No power analysis was included	Andersen et al. [31], Andersen et al. [38], Antoni et al. [7], Antoni et al. [32], Aranda et al. [5], Boesen et al. [39], Fukui et al. [40], Kissane et al. [19], Kravitz et al. [34], Rose et al. [6], Rose et al. [42].	2 Cognitive behavioral 2 Supportive 3 Group 4 Telephone

The method used for randomization and who randomized are described (1 point)	Andersen et al. [23], Aranda et al. [5], Girgis et al. [33], Kissane et al. [19], Kravitz et al. [34], Miller et al. [25], Manne et al. [35], Pitceathy et al. [26], Walker et al. [37].	2 Cognitive behavioral 1 Supportive 2 Group 4 Telephone

It is not clear how randomization was done (method and responsible individual)	Andersen et al. [31], Andersen et al. [38], Antoni et al. [7], Antoni et al. [32], Boesen et al. [39], Fukui et al. [40], Powell et al. [41], Rose et al. [6], Rose et al. [42], Spiegel et al. [36].	2 Cognitive behavioral 3 Supportive 3 Group 2 Telephone

Blinding was discussed (1 point)	Andersen et al. [31], Fukui et al. [40], Kravitz et al. [34], Pitceathy et al. [26], Walker et al. [37].	1 Cognitive behavioral 1 Supportive 1 Group 2 Telephone

Blinding was not discussed	Andersen et al. [38], Andersen et al. [23], Antoni et al. [7], Antoni et al. [32], Aranda et al. [5], Boesen et al. [39], Girgis et al. [33], Kissane et al. [19], Manne et al. [35], Miller et al. [25], Powell et al. [41], Rose et al. [6], Rose et al. [42], Spiegel et al. [36].	3 Cognitive behavioral 3 Supportive 4 Group 4 Telephone

Reasons for dropouts are given (1 point)	Antoni et al. [32], Boesen et al. [39], Fukui et al. [40], Girgis et al. [33], Kissane et al. [19], Miller et al. [25], Pitceathy et al. [26], Powell et al. [41], Spiegel et al. [36].	3 Cognitive behavioral 3 Supportive 2 Group 1 Telephone

No reasons for dropouts are provided	Andersen et al. [31], Andersen et al. [38], Andersen et al. [23], Antoni et al. [7], Aranda et al. [5], Kravitz et al. [34], Manne et al. [35], Rose et al. [6], Rose et al. [42], Walker et al. [37].	1 Cognitive behavioral 3 Group 1 Supportive 5 Telephone

An intent-to-treat analysis was completed (1 point)	Andersen et al. [31], Andersen et al. [23], Antoni et al. [7], Kissane et al. [19], Pitceathy et al. [26], Powell et al. [41], Spiegel et al. [36], Walker et al. [37].	2 Cognitive behavioral 1 Supportive 4 Group 1 Telephone

An intent-to-treat analysis is not mentioned	Andersen et al. [38], Antoni et al. [32], Aranda et al. [5], Boesen et al. [39], Fukui et al. [40], Girgis et al. [33], Kravitz et al. [34], Manne et al. [35], Miller et al. [25], Rose et al. [6], Rose et al. [42].	2 Cognitive behavioral 3 Supportive 1 Group 5 Telephone

Recruitment dates are specified (1 point)	Andersen et al. [23], Boesen et al. [39], Fukui et al. [40], Kissane et al. [19], Manne et al. [35], Miller et al. [25], Pitceathy et al. [26], Powell et al. [41], Spiegel et al. [36], Walker et al. [37].	2 Cognitive behavioral 4 Supportive 3 Group 1 Telephone

Recruitment dates are not specified	Andersen et al. [31], Andersen et al. [38], Antoni et al. [7], Antoni et al. [32], Aranda et al. [5], Girgis et al. [33], Kravitz et al. [34], Rose et al. [6], Rose et al. [42].	2 Cognitive behavioral 2 Group 5 Telephone

Study limitations are described (1 point)	Andersen et al. [31], Andersen et al. [38], Antoni et al. [7], Antoni et al. [32], Aranda et al. [5], Boesen et al. [39], Fukui et al. [40], Kissane et al. [19], Girgis et al. [33], Kravitz et al. [34], Manne et al. [35], Miller et al. [25], Pitceathy et al. [26], Powell et al. [41], Rose et al. [6], Rose et al. [42], Spiegel et al. [36], Walker et al. [37].	4 Cognitive behavioral 4 Supportive 4 Group 6 Telephone

Study limitations are not described in detail	Andersen et al. [23].	1 Group

Significant results were reported	Andersen et al. [31], Andersen et al. [38], Andersen et al. [23], Antoni et al. [7], Antoni et al. [32], Aranda et al. [5], Fukui et al. [40], Girgis et al. [33], Kissane et al. [19], Kravitz et al. [34], Manne et al. [35], Miller et al. [25], Pitceathy et al. [26], Powell, et al. [41].	4 Cognitive behavioral 3 Supportive 4 Group 3 Telephone

Significant results were not reported. Note: data collection continues for Walker [37] and Rose [6].	Boesen et al. [39], Rose et al. [42], Spiegel et al. [36].	1 Supportive 1 Group 1 Telephone

Funding sources are listed (1 point)	Andersen et al. [31], Andersen et al. [38], Andersen et al. [23], Antoni et al. [7], Antoni et al. [32], Aranda et al. [5], Boesen et al. [39], Fukui et al. [40], Kissane et al. [19], Girgis et al. [33], Kravitz et al. [34], Manne et al. [35], Pitceathy et al. [26], Rose et al. [6], Rose et al. [42], Spiegel et al. [36], Walker et al. [37].	3 Cognitive behavioral 3 Supportive 5 Group 6 Telephone

A trial registry is mentioned (1 point)	Girgis et al. [33], Kravitz et al. [34]	2 Telephone

Nurse-delivered treatment. Seven studies included nurses in providing the intervention.	(i) Coled by a nurse, physical therapist, chaplain, or social worker (Miller-CB, [25])	
	(ii) One nurse, one social worker (Pitceathly-CB, [26])	
	(iii) 3 nurses provided the intervention (Fukui-S, [40])	
	(iv) Breast cancer nurses (Aranda-T, [5])	
	(v) Masters prepared psychiatric nurses (Rose-T, [6] and S/T, [42])	
	(vi) Cancer nurses (Walker-T, [37])	

Length of study. The length of interventions varied from a single visit to one year.	Miller et al. (CB, [25]), 8 sessions/27 weeks	
	Pitceathly et al. (CB, [26]), 3 sessions/6 weeks	
	Antoni et al. (CB, [7, 32]), 2 hour sessions/10 weeks	
	Boesen et al. (S, [39]), 6, 2 hour sessions/6 weeks	
	Fukui et al. (S, [40]), interviews at baseline, 1 month, 3 months	
	Manne et al. (S, [35]), 6 sessions unclear timeframe	
	Powell et al. (S, [41]), single visit	
	Andersen et al. (G, [23, 31, 38]), 4 months weekly followed by 8 monthly for 12 months total	
	Kissane et al. (G, [19]), 1 year	
	Spiegel et al. (G, [36]), 1 year	
	Kravitz et al. (T, [34]), 1 session/telephone contact at 2, 6, and 12 weeks.	
	Aranda et al. (T, [5]), 1 session/1 telephone followup	
	Rose et al. (T, [6]), 1 session/6 weeks of telephone contact	
	Rose et al. (S/T, [42]), 2 months	
	Walker et al. (T, [37]), 8 sessions in 16 weeks and monthly telephone	
	Girgis et al. (T, [33]), telephone contact every 6 weeks for 6 months	

**Table 3:** Cognitive behavioral (CB) Interventions. Abbreviated data extraction tables are presented below to summarize each study according to type (cognitive behavioral, supportive, group, and telephone assisted). All studies were randomized controlled trials. The data extraction table corresponds to criteria from the CONSORT checklist [22].

Author (s)	Objective and hypothesis listed	Clear eligibility criteria/date study conducted	Settings and sample specified	Interventions clearly described such that replication is possible	Outcome measures specified	Sample size/power analysis	How randomization was accomplished/who randomized	Blinding	Drop out reasons specified	Limitations discussed	Funding source/trial registry	Findings
Antoni et al., [32]. Randomization not indicated in title.	Women in the cognitive behavioral stress management will report reduced anxiety and have reduced serum cortisol and cytokine production.	Female breast cancer patients with stage 1–3 who were within 4 to weeks of surgery. Specific dates for recruiting participants were not specified.	Dade county, Florida. 127 initially, 97 completed the study, 85 assays were completed. This sample came from the larger Antoni [7] study.	Interventions were clearly described including (1) a 10-week group cognitive behavioral stress management or (2) 1 day psychoeducational control group consisting of the same information without group support.	Cancer specific and general anxiety, impact of events scale, serum cortisol, Th1 and Th2 cytokine production.	No power analysis mentioned	Not discussed.	Not mentioned.	Reasons for dropping out not mentioned. Those who dropped out did not differ from completers on outcome or demographic variables. Completer analysis rather than intent-to-treat analyses were used.	Single serum samples of cortisol were used rather than collection across the diurnal cycle. The participants were middle class, white, and well educated.	No discussion of trial registry although it is likely as the study was funded by the National Cancer Institute.	The CB intervention resulted in lower anxiety, lower cortisol, and greater cytokine production.

Antoni et al., [7]. Randomization not indicated in title.	The stress management group will decrease intrusive thought over the short-term and at the end of treatment.	Female breast cancer patients with stage 1–3 who were within 4 to weeks of surgery. Specific dates for recruiting participants were not specified.	Dade county, Florida. 99 women participated	The same interventions as Antoni [32]. They were clearly described.	Thought intrusion and avoidance as measured by the impact of event scale, interviewer rated anxiety, emotional distress measured with the Affects Balance Scale.	No power analysis mentioned	Not discussed	Not mentioned.	Not mentioned. Attrition did not differ by condition. However Hispanic and younger women were more likely to drop out. An intent-to-treat analysis was used.	The sample was middle class, well educated and primarily white.	No discussion of trail registry although it is likely as the study was funded by the National Cancer Institute.	The CB intervention resulted in a greater reduction in cancer-specific thought intrusion, anxiety and emotional distress than did the control.

Miller et al., [25]. Randomization not indicated in title.	The social work (SW) component of a multidisciplinary intervention will improve quality of life of cancer patients.	A vague eligibility criterion of having “advanced cancer and receiving radiation treatment” (p. 109) was used although the authors made reference to the parent study which contained a more detailed methodology section. Recruitment dates were specified.	The setting was not described, although it was likely done in the Mayo Clinic.	An 8-session multidisciplinary intervention (90 minutes) including cognitive-behavioral strategies or standard care extending over 4 weeks. The intervention provided by the social worker was described although other aspects of the multidisciplinary intervention and the standard care interventions were not.	Quality of Life measured at baseline 4, 8, and 27 weeks using the Spitzer QOL Uniscale and Linear Analogue Self-Assessment scales.	115 of 418 eligible patients were included. A power analysis was discussed.	Patients were externally randomized by the cancer center randomization unit using the Pocock and Simon balance scheme.	No mention was made of blinding of participants or providers.	Numbers of individuals who dropped out and reasons for that were provided. No mention is made of an intent-to-treat type of analysis.	No mention was made whether other cointerventions were monitored, how treatment fidelity was determined. Cultural diversity was lacking.	No mention was made of external funding for the study or registration of the trial.	QOL at week 4 averaged 10 points higher in the intervention group (3% increase from baseline and a 9% decrease in the control group). Significant changes were seen in areas of financial concerns and legal issues (SW component) by week 4.

Pitceathly et al., [26]. The title specified randomization.	A brief intervention, delivered by nonspecialists is superior to usual care in preventing anxiety and depression.	Eligible patients were 18 to 70, newly diagnosed with a first episode of cancer and without anxiety or depressive disorders. Recruitment date years were specified.	Clinics associated with a regional cancer center in Manchester, England. 313 patients participated.	Three immediate sessions of CBT or delayed intervention at 8 weeks from diagnosis or usual care. The first session (90 minutes) was in person followed by two 45-minute telephone sessions. Interventions could be replicated.	Assessment of anxiety and depression at 6 and 12 months (Structured Clinical Interview for DSMIII). Hospital Anxiety and Depression Scale. A 14-item checklist regarding cancer-related concerns.	A power analysis was included.	Independent randomization via computer was used.	Whether patients were blinded is not specified. Data collectors were blinded.	Reasons for dropping out were specified. Analysis was by intent-to-treat.	Years of experience for the nurse and social worker or credentials were not described. Results were not analyzed to see if the type of practitioner mattered. Training and supervision were provided but not by an individual uninvolved in the study. A training manual was developed after the study concluded. At 6 months 27% of patients in the intervention and 16% in usual care could not be assessed.	The study was funded by the United Kingdom (UK) Cancer Research branch. It was not clear if the UK has trial registry.	High-risk patients who received the intervention were less likely to become depressed or anxious than those in the usual care arm at 2, 4, and 6 months. There was no difference between early and delayed intervention.

**Table 4:** Supportive interventions. Boesen et al. [39] conducted a regression analysis using an intervention that could be classified as both a supportive and group intervention.

Author (s)	Objective and hypothesis listed	Clear eligibility criteria/date study conducted	Settings and sample specified	Interventions clearly described such that replication is possible	Outcome measures specified	Sample size/power analysis	How randomization was accomplished/who randomized	Blinding	Drop out reasons specified	Limitations discussed	Funding source/trial registry	Findings
Boesen et al., [39]. The title specified randomization.	A hypothesis was not included.	Clients age 18 to 70 with cutaneous malignant melanoma of stage I and II. Recruitment dates were listed	Plastic surgery departments throughout Denmark. 262 patients participated.	Details of the intervention and the control group are described in another publication [43]. The intervention could not be replicated without referring to that article [43].	POMS, dealing with illness coping inventory, the Barret-Lennard Relationship Inventory and the Marlow-Crown scale of Social Desirability.	A power analysis was not included. 51 individuals who declined to participate in the randomized study completed in 2005 [44] were asked to fill out a baseline questionnaire and compared to 259 individuals who participated in that 2005 randomized study.	Details were not provided about the type of randomization or the random allocation sequence.	No mention is made of whether participants, providers, or data collectors were blinded.	Reasons for nonparticipation were given.	Only 40% (*N* = 51) of those who declined completed a baseline questionnaire. Who provided the group interventions and how or if treatment fidelity was monitored are not described. Ethnic and racial backgrounds were not reported.	The Danish Cancer Society funded the grant but since it was a secondary analysis, not a randomized trial itself, no mention is made of inclusion in a registry.	Higher socioeconomic status, higher coping, lower social support, and lower mood predicted participation rather than tumor characteristics. Social desirability did not predict participation.

Fukui et al., [40]. The title specified randomization.	A hypothesis was not include.	Clients over 18 with gastric, colorectal, or breast cancer not in an advanced stage. Recruitment dates were provided.	One Japanese cancer center. The background of the nurses was well described. 89 patients (51%) participated.	The intervention is not described in sufficient detail to replicate the study. A waiting list control was used.	Hospital Anxiety and Depression Scale, Mental Adjustment to Cancer Scale administered at 1 week, 1 month, and 3 months after diagnosis.	A power analysis was not included.	The method of randomization is not described in detail. Both nurses in the intervention group who received training and in the control group participated in randomizing patients. Clients were randomly assigned to trained or nontrained nurses and usual care treatment.	Clients were blinded to assignment. Nurses were not blinded.	Reasons for dropouts were discussed. No differences were found based on RN or MD characteristics. RN interviews with intervention clients were longer (22 minutes versus 18 in the control). No mention is made of intent-to-treat analysis.	Only 4 nurses were assigned to the intervention and 4 to the control group. In 24% of interviews nurses did not follow the second step of the intervention, 29% did not follow the 3rd step and 22% did not follow the 4th step indicating poor treatment adherence. Treatment fidelity was determined by audiotaping the intervention group only. Neither interrater reliability nor intrarater reliability was confirmed. The influence of physician communication was not assessed	The Japan Society for the Promotion of Science and a Pfizer Grant supported the study. No mention is made of whether a trial registry was used.	Group by time decreases in psychological distress, increases in fighting spirit, and decreases in fatalism occurred in the intervention group.

Manne et al., [35]. Randomization was not indicated in title.	The effects of coping and communication enhancing interventions on depressive symptoms would be primarily mediated by the process that were encouraged by increasing expression of emotions, increasing attempts to understand emotional reaction and improvements in self-esteem.	Eligible patients were women undergoing active medical treatment diagnosed with primary gynecological cancer, 18 or older with a Karnofsky performance status of 80 or greater living within a 2-hour older that were English speaking. Recruitment dates were listed.	10 hospitals in the northeast United States. 353 women participated.	The Coping and Communication Enhancing Intervention (CCI) was not described in sufficient detail in this publication to replicate that intervention.	The Beck Depression Inventory, COPE, Emotional Expressivity Questionnaire, the Positive Emotion Scale, Emotional Expression Scale, Rosenberg Self-Esteem Inventory evaluated at baseline, 3, 6, and 9 months.	A power analysis was completed	The statistician created the randomization scheme based on the baseline Beck Depression score and the research assistant assigned participants.	Research assistants, participants, and interventionalists were not blinded.	Reasons for drop-outs were not detailed in this publication.	Changes in depressive symptoms may have predated the intervention. The sample consisted primarily of Caucasian women. 58% declined to participate. Therapist background and training and treatment fidelity were not discussed.	No discussion of trail registry although it is likely as the study was funded by the National Cancer Institute.	The coping and supportive counseling interventions both had a beneficial effect on depressive symptoms. There was no impact of any intervention on cancer-specific distress.

Powell, et al., [41]. The title specified randomization.	The study purpose was discussed but the hypothesis was not mentioned.	The only eligibility criteria listed were having attended a gynecological cancer clinic for the first time. It was unclear if the women were actually diagnosed with cancer. Recruitment dates were specified.	A gynecological cancer clinic in San Francisco. 100 women with gynecological cancer were eligible. 43 in the control group completed questionnaires, 21 women received the intervention. The sample was primarily Caucasian (71%).	The intervention, a one-time meeting with a psychologist or a control group was not described in enough detail to replicate.	Functional Assessment of Chronic Illness Therapy, Version 4; POMS, Index of Coping Responses and satisfaction with the clinic.	The sample size was small (*n* = 21) and no power analysis was provided.	Randomization (random numbers in sealed envelope) was discussed but not who was responsible for the randomization.	Neither the patients nor the psychologist were blinded.	Reasons for nonparticipation and drop out were discussed. An intent-to-treat analysis was performed.	Only a small sample of 21 women actually received the intervention. Treatment fidelity was not discussed.	No sources of funding or trial registry were mentioned.	Women who received the intervention showed greater decreases in anxiety, depression, and distress as well as increasing physical, emotional, functional, and overall well-being.

**Table 5:** Group interventions. Three studies by Andersen et al. [23, 31, 38] were with the same sample but differing analyses were conducted at varied endpoints of the research.

Author (s)	Objective and hypothesis listed	Clear eligibility criteria/date study conducted	Settings and sample specified	Interventions clearly described such that replication is possible	Outcome measures specified	Sample size/power analysis	How randomization was accomplished/who randomized	Blinding	Drop out reasons specified	Limitations discussed	Funding source/trial registry	Findings
Andersen et al. [31]. Randomization was not indicated in title.	The intervention would have a direct positive effect on health (psychological distress, immune function, performance status)	Patients with stage II or stage II breast cancer aged 28 to 84.	A cancer center in Ohio. 227 patients participated.	Patients were assigned to an assessment only (113) or intervention plus assessment arm (114). Patients were also randomized to chemotherapy or radiotherapy. Group sessions were led by female clinical psychologists and consisted of 18 weekly sessions followed by 8 monthly groups (for a total of 1 year).	Impact of Events Scale, POMS, immune measures, Karnofsky Performance Status, symptomatology collected at baseline, 4 months, and 12 months.	No power analysis was included.	Randomization details are discussed in a different publication.	A research nurse blinded to study conditions conducted a health interview with each patient.	Reasons for dropping out were not detailed in this publication. An intent-to-treat analysis was done.	The sample was primarily Caucasian. Little discussion is directed to the study limitations.	Trial register is likely as the study was funded by the National Cancer Institute, the National Institute of Mental Health, the American Cancer Society, and the US Army Medical Research Acquisition Grant, among others.	Functional status increased by 7% in the intervention arm and by 1% in the assessment arm. Symptoms increased by 29% in the assessment arm compared to 14% in the intervention arm. For patients with high cancer-related stress at baseline, declines in mood disturbance were greater in the intervention than the assessment arm.

Andersen et al. [38]. The title specified randomization.	No hypothesis was included in this article.	Patients with stage II or stage II breast cancer aged 28 to 84. Specific recruitment dates were not specified.	A cancer center in Ohio. 227 patients.	A clearly specified intervention with a treatment manual to assure treatment fidelity was used. There was an assessment only (113) or intervention plus assessment arm (114). Patients were also randomized to chemotherapy or radiotherapy. Group sessions were led by female clinical psychologists and consisted of 18 weekly sessions followed by 8 monthly groups (for a total of 1 year).	Likert measures of participant satisfaction and group cohesion were used. In addition the POMS, the Perceived Social Support Scale for Family, the Food Habits Questionnaire, the 7 day physical activity log, the Karnofsky Performance Status Scale of physical functioning, and chemotherapy dose intensity were measured.	Secondary analyses were done. A power analysis was not included.	Randomization details were not included in this article.	Within this article blinding is not discussed.	Reasons for drop out were not specified in this article.	The sample was 90% Caucasian, 9% African American, and 1% Hispanic. Efficacy regarding individual interventions was not possible as social support, stress reduction, and improving mood for example were all included in the group sessions.	The same as Andersen et al. [31].	Clients were satisfied with the group. Reductions in emotional distress, increases in social support, dietary improvement, reduced variability in chemotherapy dose, improved immunity, fewer symptoms and higher functional status occurred with the group sessions. The intervention did not affect exercise.

Andersen et al. [23]. The title specified randomization.	The psychological intervention would after 11 years reduce risk of disease recurrence and improve survival.	Women diagnosed with breast cancer stage IIA and IIB who were surgically treated and awaiting adjuvant therapy. Recruitment dates were specified.	The same as Andersen et al. [31]. 227 patients participated.	The same as Andersen et al. [31].	Assessments occurred every 6 months in years 2 to 5 then yearly. Outcomes were recurrence-free survival, breast cancer-specific survival.	The trial was powered to detect a doubling of time to an endpoint requiring 27 events in each treatment arm.	Randomization was according to lymph node status, tumor size, hormone receptor status, menopause status, marital status. Wite and Freedman's minimization method was used to randomize. Who conducted the randomization was not described.	This article did not mention blinding.	Patients who were lost to followup had their recurrence data censored at the time of last contact. An intent-to-treat analysis was used. Intervention patient, whether they participated or not were included in the analysis.	Little time is spent in the article outlining limitations of the study.	The same as Andersen et al. [31].	After 11 years patients in the intervention arm had a reduced risk of breast cancer reoccurrence and death compared to those in the assessment arm. Median time to recurrence was 2.8 years for the group and 2.2 years for the assessment only arm. Intervention arm clients survived 6.1 years versus 4.8 years in the assessment arm.

Kissane et al. [19]. The title specified randomization.	The study was not introduced with a hypothesis.	Women with advanced breast cancer (stage IV). Recruitment dates were specified.	Seven public hospitals in Melbourne, Australia. 485 women.	Standardized training and supervision was provided to ensure treatment fidelity. 1 year of clearly defined weekly supportive group therapy was offered by psychologists, psychiatrists and social workers or 3 relaxation therapy sessions.	Survival, depression (Monash Interview for Liaison Psychiatry), quality of life (Quality of Life C-30 questionnaire), Impact of Event Scale and Minimental Adjustment to Cancer Scale.	A power analysis was not presented. A secondary analysis was conducted.	Randomization was by independent adaptive biased coin design.	There was no blinding of patients or therapists.	Reasons for drop out and refusal were given and results were analyzed by intent-to-treat.	Only 47% of eligible patients consented. Results were not reported based on differences in training of therapists nor were the numbers in each group or years of experience presented. It was not discussed whether women practice relaxation at home between sessions.	It is unclear whether there was a trial registry. The study was funded by grants from the National Health and Medical Research Council of Australia, the Cancer Council of Victoria, and the Kathleen Cuningham Foundation.	Group therapy did not prolong survival (the primary outcome) but it prevented and minimized depressive disorders, reduced helplessness, trauma symptoms, and improved social functioning.

Spiegel et al. [36]. The title specified randomization.	Treatment subject would live longer than control subjects.	Women with metastatic and/or recurrent breast cancer who were able to speak English. Recruitment dates were specified.	Kaiser and a university medical center referral in 3 San Francisco bay area communities. 125 women.	It would be difficult to replicate this study. 64 women were randomized to the weekly supportive-expressive group/education session offered by a psychiatrist, psychologist, or social worker. 61 women were randomized to the educational materials group including a 1-year health library membership.	Time to death.	A power analysis was included.	Although a computer-assisted randomization based on a biased coin design was used, the project director and a research nurse conducted the randomization.	Individuals conducting the randomization were not blinded. Women were not blinded.	Reasons for drop out and refusal were given. Analysis was by intent-to-treat.	Different professional led the groups at each site. Attendance at group sessions varied from 1 to 12.5 years. Women in the control group participated in more cancer groups outside the study. There were baseline differences across sites in age, education, hours worked.	Trial register is likely as the study was funded by the National Cancer Institute, the National Institute of Mental Health, the American Cancer Society, and the Mac Arthur foundation the Fetzer Institute.	The treatment and controlled interventions did not affect survival time. A post hoc analysis revealed estrogen receptor negative clients assigned to the group sessions lived longer than control group clients.

**Table 6:** Telephone Assisted Interventions.

Author (s)	Objective and hypothesis listed	Clear eligibility criteria/date study conducted	Settings and sample specified	Interventions clearly described such that replication is possible	Outcome measures specified	Sample size/power analysis	How randomization was accomplished/who randomized	Blinding	Drop out reasons specified	Limitations discussed	Funding source/trial registry	Findings
Kravitz et al. [34]. Randomization was not indicated in title.	The intervention would improve pain severity, pain-related impairment, and functional status.	English-speaking adults age 18–80 with lung, breast, prostate, head/neck, esophageal, colorectal, bladder, and gynecologic cancer who reported a score of 4 or more on a pain scale that at least moderately interfered with functioning.	3 health systems (Veterans, Kaiser-Permanente, and UCD) and one private practice in Sacramento, Calif. 265 patients	Treatment fidelity was maintained by training, regular reinforcement, and review of audio recordings of patient encounters. The intervention was described in detail and a treatment manual is available from the authors.	Pain severity, Medical Outcomes study pain impairment scale, physical and mental health component of the SF-12, perceived efficacy in patient-physician interaction scale, pain management subscale of the chronic pain self-efficacy scale.	A power analysis was not provided.	Computer-generated lists and a blocked randomization scheme were used to assure balanced assignment within physicians and to preserve concealment.	Patients were blinded to intervention until after signing consent. Research assistants collecting follow-up interviews and physicians were not aware of patient assignment.	Reasons for drop out were included.	Usual care was not used as the control condition rather enhanced usual care was. The study population was heterogeneous in terms of baseline pain and disease status. Because randomization occurred at the patient not physician level, physicians may have applied what they learned with one patient to other patients. Multiple patient outcomes were investigated with no correction for multiple comparisons.	Support was provided by the American Cancer Society Research Scholars Grant and the National Center for Research Resources. No mention is made of a trial registry.	Lay administered tailored education and coaching resulted in increased pain communication self-efficacy and improvement in pain-related impairment at 2 weeks but not at 6 or 12 weeks. No improvement in pain severity was seen.

Aranda et al. [5]. The title specified randomization.	Patients in the intervention group will report a decrease in psychological and informational needs and an increase in quality of life compared to women in the usual care group.	A somewhat vague intake criterion of a new diagnosis of breast cancer at an advanced stage, recurred or progressed in the preceding 12 months was used. No recruitment dates were listed.	In the outpatient clinics of 4 large urban hospitals (3 public, 1 private) in Melbourne, Australia. 105 women.	A clearly described, nurse delivered face-to-face visit (1 hour) followed by a phone call. The FOCUS framework including family involvement, optimistic attitude, coping effectiveness, uncertainty reduction, and symptom management was used. The control group received standard care.	The European Organization of Research and Treatment of Quality of Life version 2 and Supportive Care Needs Survey at months 1 and 3 after recruitment.	A power analysis was done retrospectively only showing a 70% likelihood of finding significance. The authors mention a need for a larger sample size.	Cards were shuffled and placed in consecutively numbered sealed envelopes.	No mention is made of blinding.	No reasons other than death were given for lost patients.	Although the 4 nurses were employed at different hospitals and all received the same training, there was no discussion of how fidelity to the intervention was maintained. More patients in the intervention arm were undergoing radiation. The one-week separation between the meeting and telephone call was too short to address concerns that involved meeting with other professionals.	It is unclear whether the trial was registered. It was funded by the Melbourne Breast Care Consortium, Victorian Department of Human Services, Australia.	No differences were found until the groups were divided into low and high needs. Women in the intervention group with high needs had more of a decrease in psychological needs than those in the lower need group at 1 month. No differences existed at 3 months.

Rose et al. [6]. Randomization was not indicated in title.	No hypothesis was provided.	Stage IV or stage III lung or pancreatic cancer patient (*n* = 161). Recruitment dates were not listed although recruitment is ongoing.	Two ambulatory care cancer clinics providing care to the underserved including a Veteran's Administration facility.	A coping and communication support intervention that was clearly described was offered by master's level mental health nurses. It consisted of a home visit that included a family member followed by telephone contact based on participant preference. Contact was available 24 hours/day, 7 days/week. One follow-up call was placed within 2 weeks. If individuals scored 4 or > on the distress thermometer they were called monthly.	Data was collected at 6 weeks using the POMS (profile of mood scale), health information processing style (Miller Monitoring-Blunting Style Scale), the 13-item symptom distress scale, the distress thermometer.	No power analysis was discussed.	The method of randomization and who conducted it were not addressed.	Blinding was not addressed.	Reasons for dropping out were not specified.	Each mental health nurse had a caseload of 80–100 individuals and no access to their medical record. It may have been difficult to keep track of each individual. Most participants were male.	Trial register is likely as the study was funded by the National Cancer Institute and the American Cancer Society.	Three follow-up telephone calls in 6 weeks were the norm with the nurse initiating contact. More middle-aged individuals raised concerns about communicating with family/friends. Older individuals had more comorbidity. Communication preferences between middle and older groups were similar.

Rose et al. [42]. Randomization was not indicated in title.	No hypothesis was listed.	Diagnosed with late-stage cancer (III or IV) in the last year receiving treatment at an ambulatory clinic. No recruitment dates were mentioned.	2 ambulatory cancer clinics in Cleveland (Metro Health Medical Center or Veterans). 109 younger and 101 older patients.	Usual care control or a coping and comm-unication support intervention provided 24/7 by mental health nurses who offered an in-home visit and followup. 75% of patients identified a family caregiver who also participated. Listening, validation, and education were provided in the intervention group. No mention was made of treatment fidelity. Without additional details the intervention would be hard to replicate	Data was collected at 2 months on sociodemographic data including income, well-being, depressed mood, anxiety, health information processing style, and family discord in communication.	A power analysis was not included.	The method of and who was responsible for randomization were not discussed.	No mention was made of blinding.	No reporting was provided about the number of individuals who declined to participate or reasons for declining. Intent-to-treat analysis was not mentioned.	Reliability and validity and names of specific outcome measures were not summarized. The control group was not described in detail.	Trial register is likely as the study was funded by the National Cancer Institute, Veterans Health Affairs Research, the American Cancer Society, and the A National Institute on Aging Grant.	Middle-aged patients averaged more communication support contacts than older clients. African American patients reported more family discord in communication.

Walker et al. [37]. The title specified randomization.	Supplementing usual care with the intervention will improve depressive symptoms, functioning, quality of life, satisfaction with depression care over 8 months.	Lung cancer patients with a diagnosis of major depression of at least 4 weeks. Recruitment timeframes were listed.	Multicenter trial involving outpatient clinics in Scotland. 200 patients participated.	Cancer nurses under the supervision of a psychiatrist were randomized to usual care or depression care. Six to 8 in-person sessions will be provided followed by telephone contact every 4 weeks for those in the depression care group. The usual care group was not described in sufficient detail	Depression severity (Symptom Hopkins Checklist) collected every 4 weeks over 32 weeks, severity of anxiety (Hospital Anxiety and Depression Scale), pain and fatigue, quality of life, cost of care, and satisfaction with depression care.	The study is powered at 90%	A secure computerized central randomization system and a secure web interface will be used for randomization.	Blinded data collection and analysis are planned.	An intent-to-treat analysis is planned.	Only 10% of recordings and treatment notes of nurses are compared to the treatment manual to evaluate nurse adherence to the protocol.	The trial is registered. Funding was obtained from Cancer Research in the United Kingdom.	Outcome data will be collected until June 2011 at 4, 8, 12, 16, 20, 24, 28, and 32 weeks from randomization.

Girgis et al. [33]. Randomization was not indicated in title.	Patients assigned to either intervention group will report decreased levels of anxiety, depression, and unmet supportive care needs over time in combination with improved physical and emotional functioning.	Notification by the New South Wales Central Cancer Registry of nonlocalized breast or colorectal cancer within 6 months of diagnosis, English speaking, age 18 or older. Recruitment dates are not specified.	New South Wales. 356 patients.	Interventions included usual care, a telephone caseworker (oncology nurses), and an oncologist/general practitioner model. The interventions were not described with sufficient detail for replication.	Anxiety, depression (Hospital Anxiety and Depression Scale), quality of life 30-item EORTC QOL questionnaire), perceived needs (Supportive Needs Survey), and perceived improvements needed in comm-unication with health care providers.	A power analysis was completed.	A computer-generated algorithm was used for randomization.	No mention was made of blinding.	Reasons for dropouts and declined were presented.	Participants were recruited 6 months after diagnosis and may have already adjusted psychologically. A large number of individuals declined to participate. Less than 50% of general practitioners returned data collection instruments.	No funding was mentioned. The trial was included in a registry.	No overall intervention effect was observed. Telephone counseling was more likely to have identified issues of need discussed, referrals made and strong agreement the intervention helped improve communication with health providers.
